# Panton-Valentine Leukocidin and concurrent respiratory viral infection as risk factors for fatal *Staphylococcus aureus* bacteremia

**DOI:** 10.3389/fmicb.2025.1719387

**Published:** 2026-02-04

**Authors:** Liting Zhou, Shiqi Guo, Ting Zhang, Ruru Bi, Qingzhen Han, Qiang Guo

**Affiliations:** 1 Center of Clinical Laboratory, The Fourth Affiliated Hospital of Soochow University (Suzhou Dushu Lake Hospital), Suzhou, China; 2 Department of Emergency, The Fourth Affiliated Hospital of Soochow University (Suzhou Dushu Lake Hospital), Suzhou, China; 3 Institute for Critical Care Medicine of Soochow University, Suzhou, China

**Keywords:** bacteremia, hyper-virulent ST22, Panton-Valentine Leukocidin, respiratory viral infection, *Staphylococcus aureus*

## Abstract

**Background:**

*Staphylococcus aureus* bacteremia (SAB) carries significant mortality. We sought to define clinical and pathogen-specific risk factors to guide early intervention.

**Methods:**

We conducted a retrospective cohort study of all SAB cases at our institution (Jan 2021–Mar 2025), expanding from an initial analysis of four fatal cases. Patient profiles, clinical characteristics and microbiological features were analyzed. Independent risk factors for mortality were determined through multivariate logistic regression.

**Results:**

Among 40 patients, mortality was higher in the community-associated *Staphylococcus aureus* (CASA) group (38.5% vs. 14.3%). Analysis revealed Panton-Valentine Leukocidin (PVL)-positive, methicillin-susceptible ST22 strains caused fatal outcomes in patients co-infected with influenza A or SARS-CoV-2, accompanied by severe leukopenia. Multivariate analysis identified fever before admission, PVL-positive *S. aureus* with respiratory viral co-infection, and ST22 strain infection as independent mortality risk factors. In the CASA subgroup, diabetes and PVL with viral co-infection were significant predictors.

**Conclusion:**

PVL-producing strains, especially ST22, are key mortality drivers in SAB, with effects amplified by respiratory viral co-infection. Early recognition of PVL/viral co-infection and comorbidity management are critical for improving outcomes.

## 1 Introduction

*Staphylococcus aureus* bacteremia (SAB) remains a critical global health challenge, associated with mortality exceeding 25% within 3 months despite advances in antimicrobial therapy (Bai et al., 2022). A significant clinical concern is persistent bacteremia, which occurs in a subset of patients even following appropriate antibiotic treatment. It has been suggested that prosthetic valves, septic shock, and persistent fever ≥ 48 h post-diagnosis as risk factors for SAB (De Rosa et al., 2016). While community-associated *S. aureus* (CASA) infections have been extensively characterized, emerging evidence underscores that patient outcomes are substantially modulated by the interplay between host clinical factors and pathogen virulence determinants (Tong et al., 2015). Among these virulence factors, Panton-Valentine Leukocidin (PVL) expressed by certain community-acquired strains is of particular concern. PVL is a pore-forming cytotoxin encoded by the *lukS-PV* and *lukF-PV* genes, typically integrated into the chromosome of both methicillin-sensitive *S. aureus* (MSSA) and methicillin-resistant *S. aureus* (MRSA) (Ide et al., 2025). PVL is strongly implicated in severe clinical syndromes, most notably rapidly progressive and frequently fatal necrotizing pneumonia (Loffler et al., 2013). Critically, the clinical course of PVL-associated infections may be further exacerbated by concomitant respiratory viral infections, where viral-bacterial synergism appears to accelerate disease progression (Chen et al., 2024). However, the precise synergistic associations between *pvl* expression, underlying host comorbidities, and their collective impact on clinical outcomes remain poorly characterized (Xiao et al., 2019).

We recently observed four fatal cases of SAB. Notably, three of these patients had concomitant respiratory viral infections. Genotyping analysis revealed that bloodstream isolates from all three co-infected patients carried the *pvl* gene. Clinically, these PVL-positive infections were accompanied by the rapid onset of severe leukopenia. This cluster of fatal outcomes, particularly the association of PVL-positive SAB with respiratory viral co-infection and profound leukopenia, underscores the critical need to identify specific risk factors for mortality in SAB. To systematically investigate these potential risk factors, we conducted a comprehensive retrospective cohort study, analyzing all documented cases of SAB recorded since our institution’s establishment. We characterize the clinical, microbiological and genotypic features in SAB cases. Subsequently, we employ multivariate modeling to identify independent risk factors for mortality, integrating both host variables and pathogen attributes. Our findings aim to inform targeted interventions for high-risk populations and optimize clinical management of SAB.

## 2 Materials and methods

### 2.1 Patients and data collection

**FIGURE 1:**
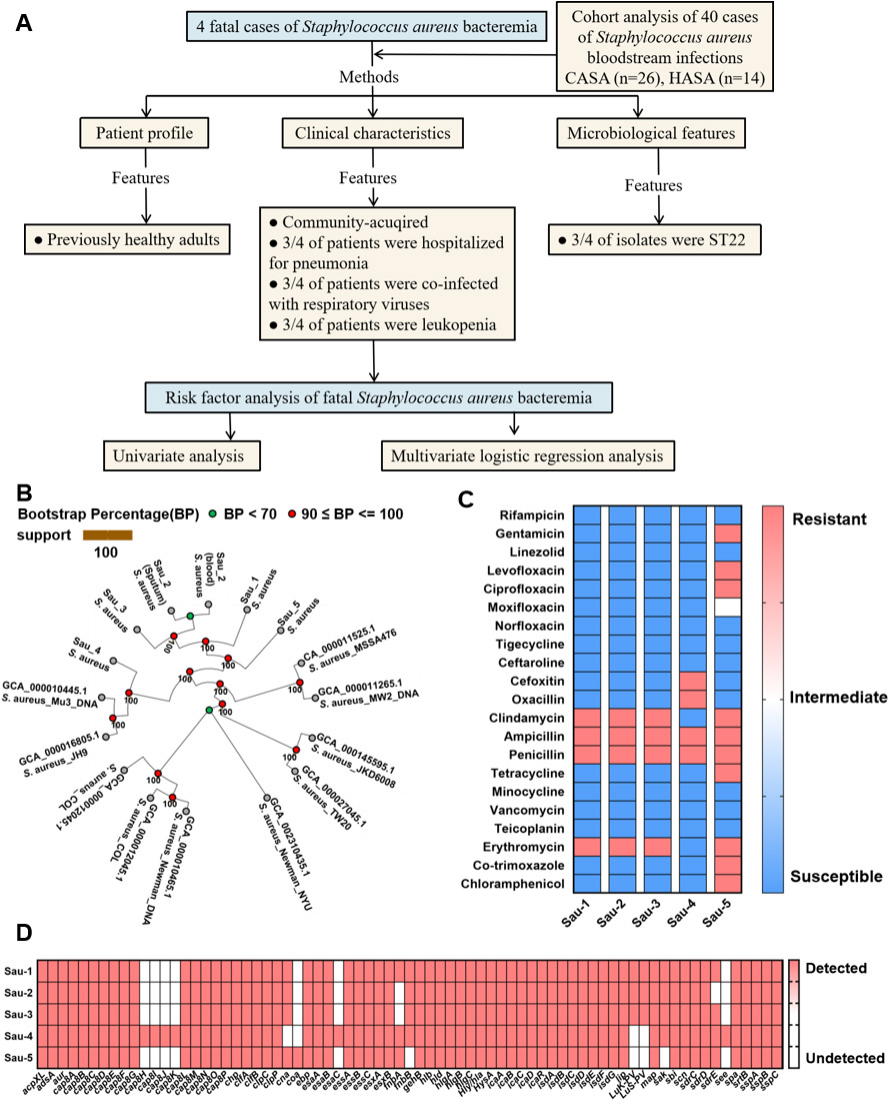
Microbiological features of 4 fatal cases of *Staphylococcus aureus* bacteremia. **(A)** Flowchart of patients enrolled for analysis. CASA: community-acquired *Staphylococcus aureus*, HASA: hospital-acquired *Staphylococcus aureus*. **(B)** Maximum-likelihood phylogenetic tree of 6 *S. aureus* bacteremia strains showing the genetic background of isolates. Of note, Sau-2 (blood) and Sau-2 (sputum) isolates were obtained from the respective clinical specimens of a single patient. The CASA strain Sau-5, isolated from a recovered patient without comorbidities, was used as a negative control. **(C)** The five *S. aureus* strains were tested for resistance to 21 antimicrobial agents by using MIC and the K-B disk diffusion method. **(D)** The virulence genes of five *S. aureus* strains were analyzed by next-generation sequencing.

This retrospective cohort study utilized data from the Fourth Affiliated Hospital of Soochow University during January 2021 to March 2025. The 40 included cases represent the complete cohort of all eligible SAB patients during this period and are not a selected subset. Inclusion criteria included: (1) definite bloodstream infection diagnosis according to standard criteria (Lamy et al., 2020) (2) at least one positive *S. aureus* blood culture, and (3) clinical evidence of infection (fever > 38°C, chills, or hypotension). Exclusion criteria were: incomplete case data, duplicate bacterial strains (retaining only the first isolate), and asymptomatic bloodstream infection. Cases were categorized as hospital-acquired or community-acquired based on *S. aureus* bacteremia (SAB) onset. Patient profiles, clinical characteristics, and microbiological features were analyzed (Figure 1A). Classification of *S. aureus* episodes was based on ECDC criteria.^1^ Hospital-acquired *S. aureus* (HASA) was defined as an isolate identified from a sample collected ≥ 48 h after admission, including those identified within 48 h if epidemiologically linked to a prior hospitalization (within 30 days). Community-acquired *S. aureus* (CASA) was defined as an isolate identified from a sample collected < 48 h after admission.

### 2.2 Criteria for prognostic evaluation

Patient prognosis was evaluated according to the following criteria. Favorable prognosis was defined as clinical recovery with normalized laboratory and microbiological findings. Non-fatal adverse outcome applied to patients who discharge against medical advice due to treatment futility and worsening clinical condition. Fatal cases included all patients who died before discharge. Poor prognosis includes both non-fatal and fatal adverse outcomes.

### 2.3 Identification and antimicrobial susceptibility testing of pathogens

Positive cultures were defined according to the Clinical and Laboratory Standards Institutes (CLSI) guidelines. Species-level identification of isolates was performed using MALDI-TOF mass spectrometry (Bruker Biotyper) following the manufacturer’s protocol. Bacterial identification and antimicrobial susceptibility testing (AST) was performed using a BD Phoenix M50 automated microbiology system (Becton Dickinson, United States) as the primary method. To ensure comprehensive resistance profiling, Kirby-Bauer (K-B) disk diffusion testing was supplemented for the following agents: Penicillin, Erythromycin, Clindamycin, Vancomycin, Linezolid, Norfloxacin, Cefoxitin, Minocycline. All AST results were interpreted in strict accordance with CLSI M100 performance standards (2024 edition).

### 2.4 Testing for respiratory viruses

Testing for respiratory viral coinfection was performed for all patients at the time of SAB diagnosis. Nasopharyngeal swab specimens were collected within 48 h of the blood culture from each patient and analyzed using a multiplex real-time polymerase chain reaction (RT-PCR) assay (Sansure Biotech, Cat# 688289), which detects a panel of common respiratory viruses including influenza A/B, respiratory syncytial virus (RSV), human rhinovirus, adenovirus, and myocoplasima pneumonia. A result was considered positive for viral coinfection if any of these targets were detected.

### 2.5 Molecular typing and virulence gene detection

Raw sequencing reads were subjected to quality control and adapter trimming using Fastp (v0.23.2) to generate high-quality clean data. *De novo* genome assembly was then performed using SPAdes (v3.15.5). The resulting draft assemblies were subsequently improved through an iterative process: gaps were filled using GapFiller, and the assemblies were polished with Pilon to correct base-level errors, including single-nucleotide polymorphisms and small insertions/deletions. For strain typing, multi-locus sequence typing (MLST) was conducted *in silico* by extracting the seven canonical housekeeping gene alleles from the polished assemblies and querying them against the *S. aureus* MLST database (PubMedST.org) to assign sequence types (STs). The presence of specific virulence genes was confirmed experimentally via conventional PCR using specific primers, followed by gel electrophoresis.

### 2.6 Statistical analysis

Data analysis was performed using IBM SPSS Statistics version 22.0. Continuous variables were first assessed for normality with the Shapiro-Wilk test. Normally distributed data are presented as mean ± standard deviation and compared using the Student’s *t*-test, while non-normally distributed data are expressed as median (interquartile range) and compared with the Mann-Whitney U test. Categorical variables are summarized as frequencies and percentages (n, %) and analyzed using the Chi-square test or Fisher’s exact test, as appropriate. Variables showing a significant association in univariate analyses (*p* < 0.05) were included in a multivariable binary logistic regression model using the enter method. Results are reported as adjusted odds ratios (ORs) with 95% confidence intervals (CIs). Model performance was evaluated in several ways: overall model significance was tested with the Omnibus test, explanatory power was quantified using Nagelkerke’s and Cox and Snell’s pseudo R^2^-values, and predictive accuracy was assessed by the overall correct classification rate based on a probability cutoff of 0.5. A two-sided *p* < 0.05 was considered statistically significant.

## 3 Results

### 3.1 Clinical course and microbiological features of 4 fatal cases of *Staphylococcus aureus* bacteremia

We collected clinical data from four patients without underlying comorbidities who were hospitalized for pulmonary infections or fever between 2022 and 2025, and subsequently developed fatal SAB. Relevant clinical parameters are cataloged in Table 1. The dynamics of inflammatory markers (white blood cells, high-sensitivity C-reactive protein and procalcitonin) throughout the infection progression are shown in Supplementary Figure S1. We found that respiratory viral co-infection was associated with significant leukopenia in 3/4 fatalities (influenza A or SARS-CoV-2 positive), whereas the virus-negative decedent exhibited preserved WBC levels. All isolates were confirmed as community-acquired *Staphylococcus aureus* (CASA), based on infections that manifested before or within 48 h of hospital admission in patients lacking recent healthcare exposure. Through spa-typing and multi-locus sequence typing (MLST), four *S. aureus* isolates clustered into two ST lineages: ST22 (3 isolates; Patients 1–3) and ST88 (1 isolate; Patient 4).

**TABLE 1:** Clinical characteristics of four fatal cases of *Staphylococcus aureus* bacteremia.

Characteristics	Variables	Patient 1	Patient 2	Patient 3	Patient 4
Patient profile	Age	34	31	73	42
	Gender	Female	Male	Male	Male
Clinical characteristics	Admission diagnosis	Pneumonia	Pneumonia	Pneumonia	Fever
	Co-infecting pathogens (site of infection)	*S. aureus*, *S*. maltophilia, Influenza A/respiratory tract	*S. aureus*, Influenza A /respiratory tract	*S. aureus*, *K*. *pneumoniae*, *P. aeruginosa* COVID-19/respiratory tract	*S. aureus*/respiratory tract
	Source of infection	Community-acquired	Community-acquired	Community-acquired	Community-acquired
	WBC	0.50∧10^9^/L	0.72∧10^9^/L	1.94∧10^9^/L	10.63∧10^9^/L
	Outcome	Death	Death	Death	Death
Microbiological features of *S. aureus*	Specimen source	blood	blood	blood	blood
	Sequence type	ST22	ST22	ST22	ST88
	Strain No.	Sau-1	Sau-2	Sau-3	Sau-4

A CASA strain, isolated from a recovered patient without underlying comorbidities, was analyzed as a negative control (designated Sau-5). An accurate determination of the genetic distance among isolates can help ascertain whether SAB is caused by the same strain or a genetically distinct strain. The maximum-likelihood phylogeny revealed distinct clustering of strains, with isolates from Sau-5 forming a monophyletic clade, while those from Sau-1 to 4 were polyphyletic across the tree (Figure 1B). As is shown in Figure 1C, 5 *S. aureus* strains isolated from 4 fatal cases and 1 recovery case underwent antimicrobial susceptibility testing against 21 agents. Sau-4 is identified as methicillin-resistant *Staphylococcus aureus* (MRSA), while the remaining isolates are methicillin-susceptible *S. aureus* (MSSA). Notably, the Sau-5 strain, classified as multidrug-resistant (MDR), was associated with a non-fatal outcome, suggesting that bacterial virulence factors can be a critical determinant of disease severity, independent of the resistance profile. All bacteria exhibit resistance to ampicillin and penicillin. Additionally, Sau-1–3 demonstrate resistance to clindamycin and erythromycin. In contrast, all strains remained susceptible to the following classes: aminoglycosides (e.g., gentamicin), oxazolidinones (linezolid), fluoroquinolones (ciprofloxacin), tetracyclines, glycopeptides (vancomycin), chloramphenicol, and neomycin-based compounds. Collectively, these results demonstrate that the 3 fatal patients were not primarily attributable to antimicrobial resistance, whereas patient 4 likely succumbed to MRSA infection. To further determine whether Sau-1 to Sau-3 represent hypervirulent *Staphylococcus aureus* (hvSA) strains, we performed next-generation sequencing of these isolates and systematically profiled their virulence determinants. Results show that Sau-1 to Sau-3 carry *lukF-PV* and *lukS-PV*, the co-transcribed genes encoding Panton-Valentine Leukocidin (PVL), and cluster with ST22. In contrast, Sau-4 lacked these virulence markers and belongs to ST88 (Table 1 and Figure 1D). Consistent with the known pathogenicity of PVL (He and Wunderink, 2020, Cheung et al., 2021), patients infected with Sau-1 to Sau-3 exhibited a sharp decrease in peripheral blood leukocyte counts (Table 1). This aligns with PVL’s ability to lyse white blood cells and cause severe necrotizing pneumonia. The absence of PVL in Sau-4 correlates with its distinct genetic lineage (ST88) and likely contributes to its divergent clinical manifestations.

### 3.2 Clinical characteristics and outcomes of patients with *Staphylococcus aureus* bacteremia

To assess the risk factors for mortality in patients with SAB, 26 Community-Acquired *Staphylococcus aureus* (CASA) and 14 HASA isolated from patients with SAB were observed during the study period. Female patients comprised 65.4% (17/26) of CASA and 71.4% (10/14) of HASA groups. HASA patients were older, though this difference was not statistically significant (67.0 vs. 56.5 years, *p* = 0.081), and had a longer previous hospital admission (34.93 ± 44.9 days vs. 15.54 ± 20.60 days, *p* = 0.0553). The distribution of CASA and HASA patients across specific clinical departments showed no statistically significant differences. The incidence of poor prognosis-defined as both non-fatal and fatal adverse outcomes was higher in CASA (38.5%, 10/26) than HASA patients (14.3%, 2/14). Fever as an admission diagnosis was more common in the CASA group (50%, 13/26 vs. 21.43%, 3/14). The CASA patients were more likely to co-infected with respiratory virus (7/26, 26.92%) than the HASA patients (2/14, 14.29%). The most common commodity is hypertension both in CASA patients (38.46%) and HASA patients (64.29%). Neither history of antibiotic use (53.8% in CASA vs. 64.3% in HASA, *p* = 0.524) nor receipt of invasive procedures (57.7% vs. 71.4%, *p* = 0.392) differed significantly between groups (Table 2).

**TABLE 2:** Comparison of different clinical characteristics between CASA and HASA patients with *S. aureus* bacteremia.

Clinical characteristics	CASA (*n* = 26), no. (%)	HASA (*n* = 14), no. (%)	χ^2^/*t*-value	*P*-value
Age	56.46 ± 18.32	67 ± 16.46	1.796	0.0805
Gender (female, no.)	17 (65.38)	10 (71.43)	0.152	0.697
Previous hospital admission (days)	15.54 ± 20.60	34.93 ± 44.90	1.977	0.0553
**Department**				
Orthopedic ward	4 (15.38)	2 (14.29)	0.009	0.962
General medicine ward	3 (11.54)	1 (7.14)	0.195	0.658
Intensive care unit	4 (15.38)	1 (7.14)	0.565	0.452
Hemodialysis clinic	2 (7.69)	1 (7.14)	0.04	0.95
Emergency internal medicine	4 (15.38)	0 (0.00)	2.393	0.122
Dermatology and aesthetic ward	1 (3.85)	1 (7.14)	0.208	0.648
Emergency ICU	4 (15.38)	3 (21.43)	0.230	0.631
General surgery ward	1 (3.85)	0 (0.00)	0.552	0.457
Infectious diseases ward	1 (3.85)	0 (0.00)	0.552	0.457
Other departments	2 (7.69)	5 (35.71)	4.949	0.026
Poor prognosis	10 (38.46)	2 (14.29)	2.533	0.112
Admission diagnosis: fever	13 (50.00)	3 (21.43)	3.095	0.079
Co-existing respiratory viral infection	7 (26.92)	2 (14.29)	0.833	0.361
**Comorbidities**				
Diabetes	5 (19.23)	3 (21.43)	0.027	0.868
Hypertension	10 (38.46)	9 (64.29)	2.434	0.119
Cerebrovascular disease	2 (7.69)	3 (21.43)	1.57	0.21
Renal insufficiency	8 (30.77)	5 (35.71)	0.101	0.75
Pulmonary disease	8 (30.77)	5 (35.71)	0.101	0.75
Cardiovascular disease	7 (26.92)	7 (50.00)	2.13	0.144
Malignant solid tumor	4 (15.38)	3 (21.43)	0.230	0.631
History of antibiotic use	14 (53.85)	9 (64.29)	0.406	0.524
Invasive procedures	15 (57.69)	10 (71.43)	0.733	0.392

### 3.3 Microbiological characteristics and sequence type studies in *Staphylococcus aureus* from bloodstream infection

Several geographically different lineages are associated with CASA infections (Vasconcelos et al., 2023). Among the bacteremia cases, 20 *S. aureus* clinical isolates were subjected to MLST. Results show ST22 was significantly associated with poor prognosis (100% of ST22 cases vs. 0% in favorable outcome, *p* = 0.01). ST88 demonstrated borderline association with hospital acquisition (HASA), comprising all 5 HASA isolates versus 5/15 CASA cases (*p* = 0.051). Other sequence types showed no significant associations, though rare STs exhibited distinct distribution patterns (Table 3).

**TABLE 3:** The STs types of *S. aureus* isolated from bloodstream.

Sequence type	No. of positive isolates	Favorable prognosis (n = 14)	Poor prognosis(n = 6)	χ^2^/*t*-value	*P*-value	CASA	HASA	χ^2^/*t*-value	*P*-value
88	10	7	3	0.22	0.639	5	5	3.810	0.051
22	3	0	3	6.554	0.01	3	0	1.513	0.219
97	2	2	0	1.197	0.274	2	0	0.952	0.329
94	1	1	0	0.567	0.452	1	0	0.451	0.502
91	1	1	0	0.567	0.452	1	0	0.451	0.502
76	1	1	0	0.567	0.452	1	0	0.451	0.502
544	1	1	0	0.567	0.452	0	1	2.456	0.117
630	1	1	0	0.567	0.452	1	0	0.451	0.502
Total	20	14	6			14	6		

The 40 *S. aureus* strains were tested for resistance to 14 antimicrobial agents by using MIC and the K-B disk diffusion method. Extreme β-lactam resistance was observed, with 82.5% (33/40) of isolates resistant to penicillin and 70% (28/40) to ampicillin. Moderate resistance rates were noted for macrolides (erythromycin: 37.5%) and lincosamides (clindamycin: 37.5%). Of the 40 *S. aureus* episodes, 22.5% (9/40) were methicillin-resistant *S. aureus* (MRSA). However all strains were sensitive to glycopeptides (vancomycin/teicoplanin: 0% resistance), aminoglycosides (gentamicin: 0%), and newer agents including ceftaroline and tigecycline (Supplementary Table S1).

As shown in Table 4, we detected genes such as toxin, invasive enzymes, surface virulence factors, and ATP-binding cassette transport protein. Virulence gene profiling revealed near-universal presence of the fibronectin-binding gene *fnbB* (90%), alongside high conservation of adhesion factors *clfB* (75%), *clfA* (70%), hemolysin *hla* (75%), and staphylokinase *sak* (75%). Toxin genes showed heterogeneous distribution, with *sea* and *sec* being co-prevalent (70% each). The immunomodulator *tst* was detected in 70% of isolates, while *pvl* was less common (20%).

**TABLE 4:** Virulence gene profiles of 20 *S. aureus* bloodstream strains.

Virulence gene class	Virulence genes	Isolates no., (%)
**Toxin**		
Enterotoxin	*sea*	14 (70)
	*seb*	6 (30)
	*sec*	14 (70)
	*sed-1*	3 (15)
	*sed-2*	7 (35)
	*see*	3 (15)
	*selk*	5 (25)
	*selq*	3 (15)
Panton-Valentine Leukocidin	*pvl*	4 (20)
Toxic shock syndrome toxin	*tst*	14 (70)
Hemolysin	*Hla*	15 (75)
Invasive enzymes		
Clumping factor	*clfA*	14 (70)
	*clfB*	15 (75)
Fibronectin-binding proteins	*fnbA*	4 (20)
	*fnbB*	18 (90)
Staphylokinase	*sak*	15 (75)
	*chuU*	11 (55)
Surface virulence factors	*spa*	14 (70)
ATP-binding cassette transport protein	*msbA*	9 (45)

**FIGURE 2:**
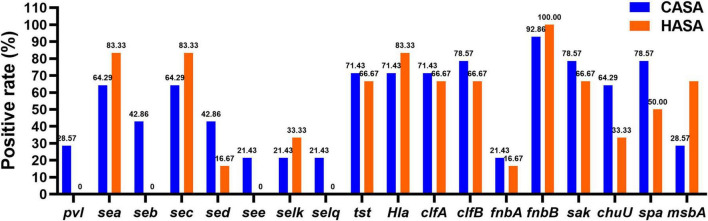
Virulence gene profiles among 20 bloodstream infection isolates of CASA and HASA.

The antimicrobial resistance profiles of *S. aureus* isolates, stratified by acquisition type (CASA vs. HASA), are presented in Supplementary Figure S2. Notably, β-lactam resistance was significantly higher in CASA isolates compared to HASA isolates. Penicillin resistance reached 96.2% (25/26) in CASA vs. 57.1% (8/14) in HASA, while ampicillin resistance was observed in 80.8% (21/26) of CASA and 50.0% (7/14) of HASA isolates. Similarly, elevated resistance rates were evident for macrolides and lincosamides among CASA isolates. Clindamycin and erythromycin demonstrated identical resistance profiles, with resistance rates of 46.2% (12/26) in CASA compared to 21.4% (3/14) in HASA. Conversely, susceptibility to several antibiotic classes remained high across both groups. Aminoglycosides (gentamicin), ansamycins (rifampin), fusidane (fusidic acid), and glycopeptides (vancomycin) exhibited minimal or negligible resistance in both CASA and HASA isolates. To further characterize pathogenicity differences, virulence gene distribution was analyzed by acquisition type (Figure 2). Strikingly, specific toxin genes (*pvl*, *seb*, *see*, *selq*) were exclusively detected in CASA isolates. Conversely, HASA isolates displayed significantly higher carriage rates of genes encoding enterotoxins (*sea*, *sec*, *selk*), hemolysin (*hla*), adhesion factors (*fnbB*), and transport proteins (*msbA*) compared to CASA isolates.

### 3.4 Risk factors for poor prognosis in *Staphylococcus aureus* bacteremia patients

The factors associated with fatal cases at univariate analysis were fever before admission, PVL with concomitant respiratory viral infection and ST22 strains (Table 5). A multivariate logistic regression analysis was performed to identify independent risk factors for mortality in *S. aureus* bacteremia (Table 6). The multivariate analysis confirmed fever before admission, PVL with concomitant respiratory viral infection and ST22 strains as the independent risk factors for mortality in *S. aureus* bacteremia patients. Since all *S. aureus* strains isolated from fatal cases were community-acquired, the CASA isolates associated with bloodstream infections were subjected to further univariate analysis. The univariate analysis indicates diabetes and PVL with concomitant respiratory viral infection were independent risk factor for mortality in CASA patients (Supplementary Table S2). Using fatal cases as the reference group, we assigned values to key variables as follows: diabetes (0 = No, 1 = Yes), PVL (Panton-Valentine Leukocidin)-positive *S. aureus* with concurrent respiratory virus infection (0 = No, 1 = Yes). The multivariate model identified diabetes mellitus and PVL-positive *S. aureus* with respiratory virus co-infection as significant risk factors for mortality (Supplementary Table S3). The model exhibited strong discriminatory power, as indicated by pseudo R-squared values (Cox & Snell = 0.806, Nagelkerke = 0.946, McFadden = 0.857) and an overall correct prediction rate of 92.9%. However, the overall model chi-square was not statistically significant (χ^2^ = 22.941, *p* = 0.115). This lack of significance is likely attributable to the limited number of fatal cases (*n* = 4) and the presence of strong, non-overlapping risk factors (quasi-complete separation). To evaluate the robustness of the identified associations despite this limitation, a post-hoc power analysis was performed using G*Power (v3.1). Under a case-to-control ratio of 6:34, observed exposure rates (50% in fatal cases vs. 0% in survivors), and α = 0.05, Fisher’s exact test revealed a statistical power exceeding 99.9%. This indicates a high probability of detecting the observed strong effects, suggesting that the associations are robust even within the constraints of a modest sample size.

**TABLE 5:** Univariate analysis of risk factors for mortality in patients with *S. aureus* bacteremia.

Risk factors	Non-fatal cases (*n* = 34)	Fatal cases (*n* = 6)	χ^2^/*t*-value	*P*-value
Age	61.26 ± 17.85	53.83 ± 20.64	0.9198	0.3635
Gender (Female, No.)	10	3	0.985	0.321
**Source of infection**				
Community-acquired	21	5	1.043	0.307
Hospital-acquired	13	1	1.043	0.307
Previous hospital admission (days)	23.62 ± 34.27	15.00 ± 14.89	0.6010	0.5514
**Comorbidities**				
Diabetes	6	2	0.784	0.376
Hypertension	17	2	0.568	0.451
Cerebrovascular disease	5	0	1.008	0.315
Renal insufficiency	11	2	0.002	0.962
Pulmonary disease	10	3	0.985	0.321
Cardiovascular disease	13	1	1.043	0.307
Malignant solid tumor	7	0	1.497	0.221
Admission diagnosis: fever	11	5	5.523	0.019
History of antibiotic use	20	3	0.162	0.687
Invasive procedures	23	2	2.562	0.109
MRSA	7	2	0.475	0.494
Concomitant respiratory viral infections	7	3	2.353	0.125
*Pvl* with concomitant respiratory viral infection	0	3	18.378	0
ST22	0	3	18.378	0

**TABLE 6:** Multivariate logistic regression analysis for mortality in patients with *S. aureus* bacteremia.

Risk factor	*β-*value	S.E.	Wald	OR value	*P-*value	95% CI
Admission diagnosis: fever	1.482	1.283	1.334	4.4	0.019	0.618–75.362
*Pvl* with concomitant respiratory viral infection	−14.667	3090.117	0	0	0.003	0.799–90.16
ST22	−32.501	4546.906	0	0	0	0.984–1.028

## 4 Discussion

This study delineates four fatal cases of previously healthy adults who was hospitalized in China for the treatment of a community-acquired MSSA necrotizing pneumonia with an underlying respiratory viral infection (influenza A/SARS-CoV-2). Supplemented by a broader cohort of 40 bacteremia patients, we revealed that PVL-producing ST22 strains drive hypervirulence in hosts, manifesting as rapid leukopenia and fatal outcomes, particularly when concomitant respiratory viral infection is present. PVL and respiratory viral co-infection emerges as an independent risk factor for mortality in both the overall SAB cohort and the CASA subgroup. Additionally, ST22 lineage is significantly associated with poor prognosis, while diabetes independently heighten mortality risk in CASA patients.

Contrary to historical perceptions, CASA infections carried a higher mortality than HASA (38.5% vs. 14.3%). This may be attributable to the exclusive presence of *pvl*^+^ strains in CASA, which typically cause severe, acute community-onset infections. Furthermore, the high mortality in CASA occurred despite universal susceptibility to glycopeptides and aminoglycosides, indicating that treatment failure was driven by virulence, not resistance. The case of the multidrug-resistant Sau-5 strain, which was associated with a non-fatal outcome, further reinforces the concept that bacterial virulence factors are a pivotal and independent determinant of disease severity.

Although disease presentation and severity of infection generally depend on combinations of virulence and host factors, some specific well-characterized virulence factors are associated with characteristic disease manifestations. The fatal trajectory of *pvl*^+^ *S. aureus* aligns with established mechanisms of PVL-mediated cytolysis: rapid neutrophil destruction precipitates leukopenia, impairing bacterial clearance and facilitating necrotizing tissue damage (Balakirski et al., 2020). Crucially, our data extend this paradigm by demonstrating that respiratory viral co-infection exacerbates PVL-driven pathology. Although the temporal sequence of pathogen acquisition cannot be definitively established, this synergistic exacerbation likely arises through several potential mechanisms: (1) virus-induced down-regulation of innate immune defenses (Feys et al., 2022); (2) viral neuraminidase exposure of host receptors (e.g., sialylated glycans) promoting *S. aureus* colonization (Wilden et al., 2021); and (3) concurrent viral and PVL-induced cytokine storms, which intrigger the uncontrolled release of neutrophil proteases into the pulmonary microenvironment, thereby driving massive tissue destruction (Loffler et al., 2013).

ST22 originated as a hospital-acquired MRSA lineage, first identified as the epidemic strain MRSA-15 (EMRSA-15) in the United Kingdom (Zhao et al., 2023). While ST22 MRSA strains only sporadically reported in China, a concerning trend has emerged: community-acquired infections caused by PVL-positive MSSA ST22 are becoming increasingly prevalent in Urumqi and across China (Yuan et al., 2019). This hyperepidemic *pvl*^+^ MSSA ST22 clade has been predominantly associated with skin and soft tissue infections (Xiao et al., 2019). Notably, our data reveal a stark concentration of poor outcomes specifically among ST22 isolates (100% mortality vs. non-ST22, *p* = 0) underscoring the heightened pathogenic potential of this lineage. This virulence contrasts with the typically lower virulence potential observed in other lineages like ST88 (Wang et al., 2022).

Diabetes mellitus substantially increases the risk and severity of *S. aureus* bacteremia (Lee et al., 2023, Vanderschelden et al., 2019). This association is mechanistically linked to the altered metabolic environment in diabetic patients. Specifically, hyperglycemia provides abundant glucose, an essential nutrient that supports the survival and proliferation of *S. aureus* within the host. Moreover, high levels of glucose-6-phosphate, a metabolite elevated in diabetes, can induce the expression of key staphylococcal virulence factors, leading to accelerated tissue damage and necrosis (Seo et al., 2021). Additionally, diabetes promotes intracellular colonization of high virulence *S. aureus* via regulation of bacterial virulence gene expression (Wang et al., 2023). Together, these mechanisms those enhanced bacterial growth, upregulated virulence, and promoted intracellular persistence synergize with diabetes-induced impairments in host immunity and microvascular integrity. This multifaceted dysfunction collectively elevates the risk of initial infection, facilitates bloodstream dissemination, and predisposes patients to severe complications such as metastatic infection and infective endocarditis.

Although our study has limitations, including the uncertain long-term outcomes of patients discharged due to futility and a multivariate model limited in generalizability by its sample size and lack of significance (*p* = 0.115), the robust effect sizes of the identified risk factors are noteworthy. Specifically, PVL with viral co-infection exhibited a dramatically high odds ratio (>50), suggesting a pivotal role in severe outcomes. This compelling association warrants validation in larger prospective cohorts to assess its utility as a clinical biomarker and demands mechanistic exploration through dedicated in vitro co-infection models.

## 5 Conclusion

Based on the comprehensive analysis of both fatal cases and a broader cohort, we conclude that PVL-producing *S. aureus* strains, particularly those of the ST22 lineage, are key drivers of mortality in SAB. The lethal potential of these strains is dramatically amplified by concomitant respiratory viral infections (Influenza A or SARS-CoV-2), leading to a rapidly progressive clinical syndrome characterized by severe leukopenia. In the CASA setting, which carries a disproportionately high mortality, diabetes mellitus further independently heightens the risk of fatal outcomes. Our findings underscore that virulence determinants and specific pathogen-host interactions, rather than antimicrobial resistance alone, are critical in determining patient survival. Early clinical recognition of the PVL/viral co-infection syndrome, coupled with aggressive intervention and meticulous comorbidity management, is paramount for improving outcomes in high-risk patients with SAB.

## Data Availability

The original contributions presented in this study are included in this article/Supplementary material, further inquiries can be directed to the corresponding author.

## Footnotes

1
https://www.ecdc.europa.eu/en
