# Supplementary material for: Panton-Valentine Leukocidin and concurrent respiratory viral infection as risk factors for fatal Staphylococcus aureus bacteremia
Source: Front Microbiol. 2026 Feb 4;16:1719387. doi: 10.3389/fmicb.2025.1719387 (PMC12913544; doi:10.3389/fmicb.2025.1719387)
Supplement: Supplementary file 1 [file Supplementary_file_1.docx]

Supplementary Material

Figure S1


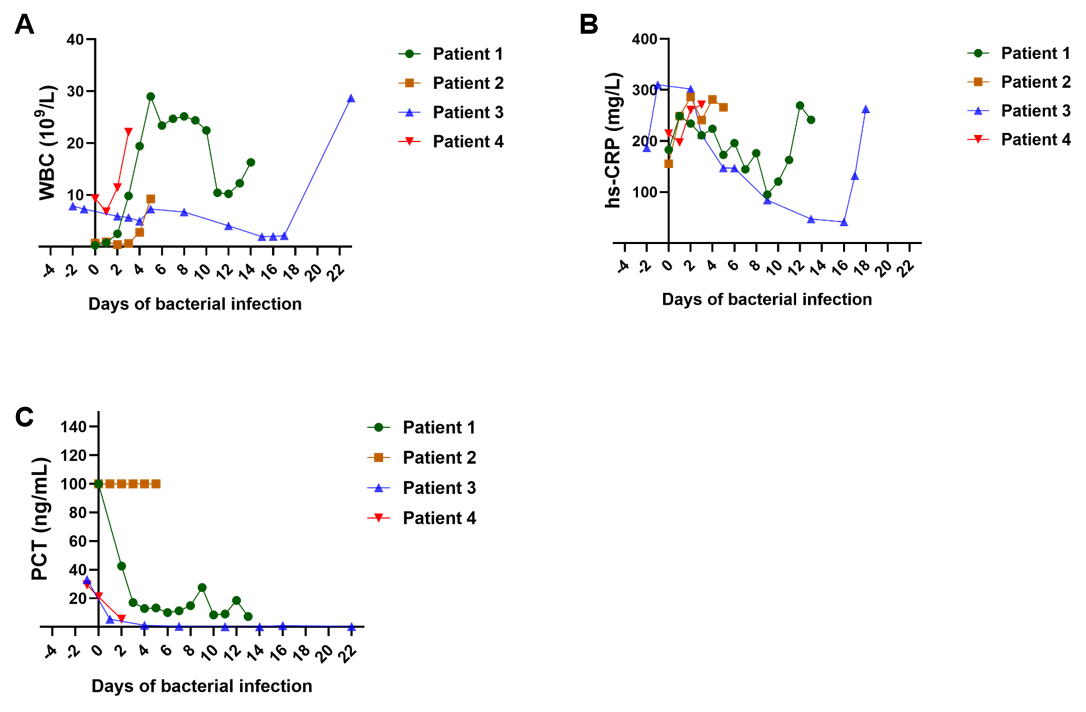
**Figure S1 The dynamics of inflammatory markers throughout the infection progression.** **(A)** white blood cells, **(B)** high-sensitivity C-reactive protein, and **(C)** procalcitonin.**Table S1 Resistance profiles of 40 *S. aureus* isolates from bloodstream infections**

| **Antibiotic class** | **Antiobiotic** | **Isolates, No, (%)** |
| --- | --- | --- |
| **Aminoglycosides** | Gentamicin | 0, (0) |
| **Ansamycins** | Rifampin | 1, (2.5) |
| **Fluoroquinolones** | Ciprofloxacin | 6, (15) |
|  | Levofloxacin | 4, (10) |
| **Fusidane** | Fusidic acid | 0, (0) |
| **Glycopeptides** | Vancomycin | 0, (0) |
|  | Teicoplanin | 0, (0) |
| **Lincosamide** | Clindamycin | 15, (37.5) |
| **Macrolides** | Erythromycin | 15, (37.5) |
| **Oxazolidinone** | Linezolid | 3, (7.5) |
| **Amphenicols** | Chloramphenicol | 3, (7.5) |
| **Phosphonic acids** | Fosfomycin | 0, (0) |
| **Streptogramins** | Quinupristin/Dalfopristin | 0, (0) |
| **Tetracyclines** | Tetracycline | 1, (2.5) |
|  | Minocycline | 0, (0) |
|  | Tigecycline | 0, (0) |
| **β-Lactams** | Penicillin | 33, (82.5) |
|  | Ampicillin | 28, (70) |
|  | Ceftaroline | 0, (0) |
|  | Oxacillin | 9, (22.5) |


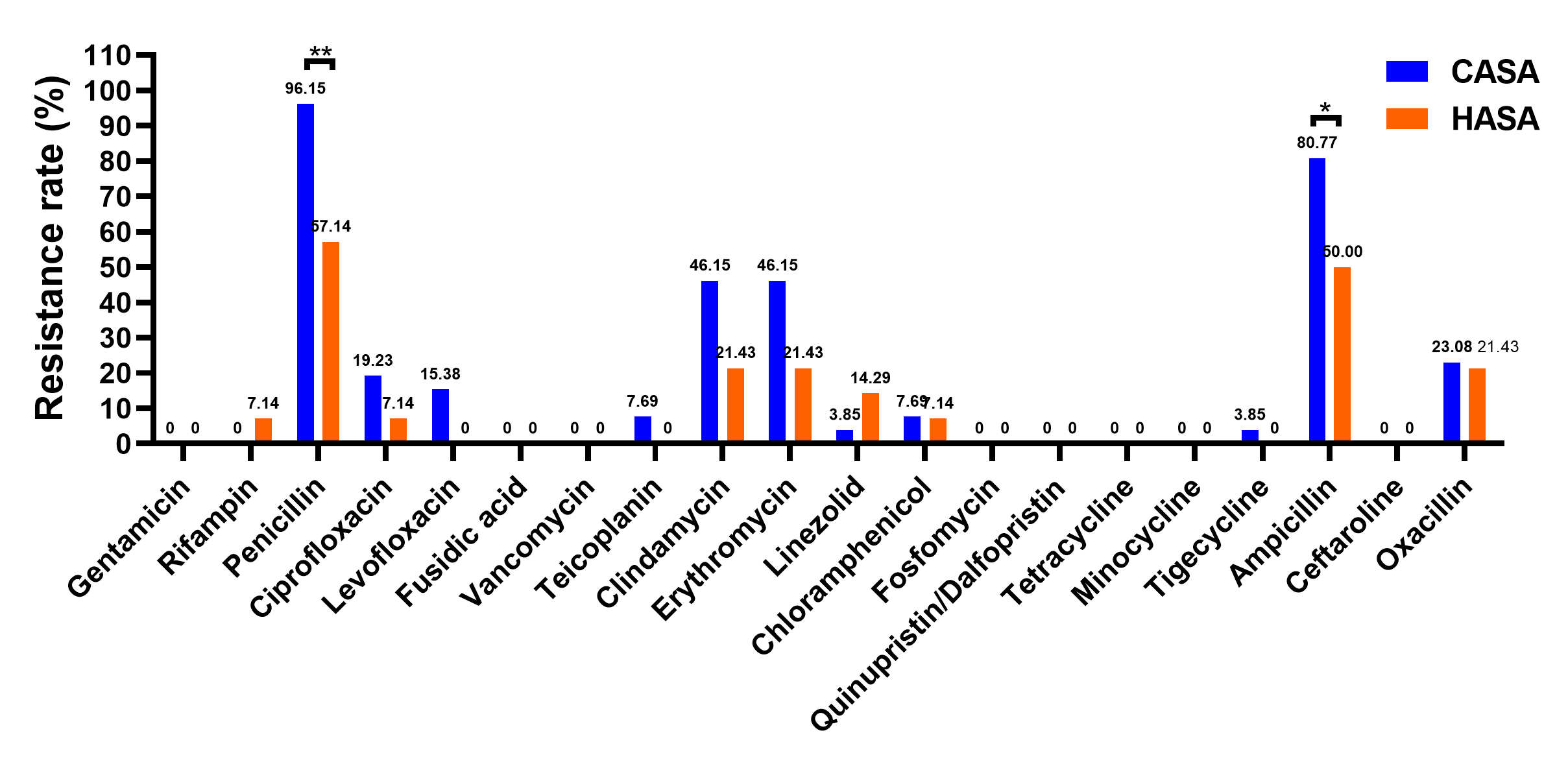
Figure S2

**Figure S2 Antibiotic resistance profiles among 40 bloodstream infection isolates of CASA and HASA**

**Table S2 Univariate analysis of poor prognosis in CASA patients with bloodstream infection**

|  | **Favorable prognosis（n=18）** | **Non-fatal adverse outcome (n=5)** | **Death**  **(n=5)** | **χ^2^/t /F value** | ***P* value** |
| --- | --- | --- | --- | --- | --- |
| **Age** | 57.13±18.75 | 61.8±19.77 | 49±18.91 | 0.59 | 0.55 |
| **Gender (Female, No.)** | 12 | 2 | 3 | 1.168 | 0.558 |
| **Previous hospital admission (days)** | 18.74±25.07 | 5.8±3.96 | 12.8±15.51 | 0.7423 | 0.4862 |
| **Comorbidities** |  |  |  |  |  |
| Diabetes | 1 | 3 | 1 | 7.927 | 0.019 |
| Hypertension | 7 | 2 | 1 | 0.657 | 0.72 |
| Cerebrovascular disease | 1 | 1 | 0 | 1.699 | 0.428 |
| Renal insufficiency | 5 | 1 | 2 | 0.506 | 0.777 |
| Pulmonary disease | 4 | 2 | 2 | 0.996 | 0.608 |
| Cardiovascular disease | 4 | 2 | 1 | 0.741 | 0.690 |
| Malignant solid tumor | 3 | 1 | 0 | 1.050 | 0.592 |
| **History of antibiotic use** | 8 | 4 | 2 | 2.222 | 0.329 |
| **Invasive procedures** | 11 | 3 | 1 | 2.760 | 0.252 |
| **MRSA** | 4 | 1 | 1 | 0.019 | 0.991 |
| **Concomitant respiratory viral infections** | 4 | 0 | 3 | 5.007 | 0.082 |
| ***Pvl* with concomitant respiratory viral infection** | 0 | 0 | 3 | 15.456 | 0 |

**Table S3 Multivariate logistic regression analysis of poor prognosis in CASA patients with bloodstream infection**

| **Prognosis** |  | **β value** | **S.E.** | **Wald** | ***P* value** | **Exp(B)** | **95% CI** | |
| --- | --- | --- | --- | --- | --- | --- | --- | --- |
|  |  |  |  |  |  |  | **Upper bound** | **Lower bound** |
| Favorable prognosis | Intercept | -39.22 | 4220 | 0 | 0.993 |  |  |  |
|  | diabetes = 0 | 3.595 | 2.049 | 3.079 | 0.079 | 36.432 | 0.657 | 2021.544 |
|  | diabetes = 1 | 0 | 0 | 0 | 0 | 0 | 0 | 0 |
|  | Pvl with concomitant respiratory viral infection = 0 | 21.167 | 3893.2 | 0 | 0.996 | 1559152897 | 0 | 0 |
|  | Pvl with concomitant respiratory viral infection = 1 | 0 | 0 | 0 | 0 | 0 | 0 | 0 |
| Non-fatal adverse outcome | Intercept | -18.9 | 1.77 | 114.03 | 0 |  |  |  |
|  | diabetes = 0 | -0.287 | 1.851 | 0.024 | 0.877 | 0.751 | 0.02 | 28.261 |
|  | diabetes = 1 | 0 | 0 | 0 | 0 | 0 | 0 | 0 |
|  | Pvl with concomitant respiratory viral infection = 0 | 19.786 | 0 | 0 | 0 | 391548538 | 391548538.4 | 391548538.4 |
|  | Pvl with concomitant respiratory viral infection = 1 | 0 | 0 | 0 | 0 | 0 | 0 | 0 |
